# Conversational technology and reactions to withheld information

**DOI:** 10.1371/journal.pone.0301382

**Published:** 2024-04-11

**Authors:** Nikolos Gurney, George Loewenstein, Nick Chater

**Affiliations:** 1 Institute for Creative Technologies, University of Southern California, Los Angeles, CA, United States America; 2 Department of Social and Decision Sciences, Carnegie Mellon University, Pittsburgh, PA, United States America; 3 Warwick Business School, The University of Warwick, Coventry, England, United Kingdom; Institute for Economic Forecasting, Romanian Academy, ROMANIA

## Abstract

People frequently face decisions that require making inferences about withheld information. The advent of large language models coupled with conversational technology, e.g., Alexa, Siri, Cortana, and the Google Assistant, is changing the mode in which people make these inferences. We demonstrate that conversational modes of information provision, relative to traditional digital media, result in more critical responses to withheld information, including: (1) a reduction in evaluations of a product or service for which information is withheld and (2) an increased likelihood of recalling that information was withheld. These effects are robust across multiple conversational modes: a recorded phone conversation, an unfolding chat conversation, and a conversation script. We provide further evidence that these effects hold for conversations with the Google Assistant, a prominent conversational technology. The experimental results point to participants’ intuitions about why the information was withheld as the driver of the effect.

## Introduction

Artificial intelligence (AI) powered conversational technology allows for information exchange via interactive communication (meaning that dialogue occurs) between a person and the technology. Commonly referred to as chatbots and virtual assistants, these digital agents are supplanting human workers across the service sector. Building on existing research, we document how conversational technology affects responses to withheld information. Comparing responses to withheld information delivered via conversational technology to information delivered in more conventional forms, we find that with conversational technology, people are more likely to infer that the information is missing for a strategic purpose and are subsequently more likely to recall its absence. We argue that these differences in interpretation of, and attention to, missing information occur because conversational technology brings into play inferential capabilities that arise spontaneously in conversational interactions.

These findings offer novel insights and have practical implications. Although the idea that conversational processing may influence cognitive processes is not new, the current research is the first to demonstrate that conversational technologies systematically alter inferences regarding missing information. This finding adds a meaningful moderator to past investigations of the impact of missing information, and suggests that using conversational technology in tests of theories from economics (unraveling theory) or consumer behavior (prompted inference-making, persuasion knowledge) might change the observed results. Further, the present findings add a critical element to prior studies of persuasion [1]: Conversational technology, relative to other modes of communicating information, may recruit different knowledge of persuasion tactics.

From a practical perspective, knowing how information delivery mode changes the inferences people draw from present or absent information is crucial to managerial and consumer decision making. This research suggests that it is risky to assume people will respond to informational content delivered via conversational technology in a fashion similar to other modes, such as print. More specifically, our results suggest that the consequence of not disclosing unfavorable information may differ substantially depending on the medium used to deliver the information.

The flip side of the same coin is that conversational technology can potentially empower people to make better decisions. The ability to make informed, accurate choices is contingent on whether a person draws the correct inferences from the information that they receive as well as information that they *do not receive*. By bringing into play abilities associated with the processing of conversations, conversational technology can lead to improved judgments and choice outcomes. Given these benefits, our research has implications for policymakers. Specifically, it suggests that policymakers interested in providing or mandating the provision of information to people should consider using conversational technology.

## Motivation

Diverse research in consumer behavior, psychology, economics, and management explores of how people form judgments when the information they receive is incomplete (e.g., [2–8]). This work makes it clear that people routinely encounter missing information, whether due to the limits of the communication medium, strategic behavior by a firm, or even their own limited attention [9]. Conversational technology represents a change in the mode of information transmission. Understanding whether and how people respond differently to limited information depending on the mode of delivery has a bearing on the safety of individuals, the fortunes of firms, and the success of politicians.

### Models of how people infer missing information

When an interested party, such as a seller, withholds complete, reliable, and potentially useful information economics has a strong prediction for how people will respond: they will assume the worst about any information that is known to exist but not provided [2, 3]. Known as “unraveling theory”, this theoretical model of behavior assumes that both parties of a transaction are rational and optimize their individual interests. In its simplest form, the theory further assumes that explicit deceit—i.e., lying—is not possible (perhaps for liability reasons) and that a business, for example, does not want a potential customer to assume that its product is lower quality than it is. Under these assumptions, the model’s logic is straightforward: Should a person encounter a product description with a missing quality measure, she will first assume that the missing information is at the average of possible values. Recognizing that a business would rationally disclose a better-than-average quality measure (because it does not want people to assume that its product is of lower quality than it is), the person updates her assumption by taking the average of the lower half of the quality distribution. The model assumes that the person will repeatedly apply this logic until she fully “unravels” the missing information and concludes it must be as bad as possible.

Although one stylized lab-based experiment supported predictions of unraveling theory [10], as did one analysis of market data [11], most evidence from economics suggests that people do not naturally engage in unraveling-like logic. Brown and coauthors [12], for example, analyzed consumer responses to cold-opened movies—that is, movies that studies do not release to critics before premiering. They found that people are insufficiently skeptical of cold-opened, hence unreviewed, movies. Comparing cold-opened movies to those of similar quality that underwent (typically lukewarm) critical review, the authors found that the cold-opened movies performed significantly better at the box office. They interpret this outcome to mean that people were failing to fully infer the extent to which a missing critical review indicated poor quality. The authors also provide anecdotal evidence that movie studios appreciate and deliberately exploit this inferential failure of moviegoers.

The field of consumer behavior provides somewhat different models of how people respond to missing information. One stream of research suggests that people do not spontaneously engage in unraveling but can and do if prompted [13, 14]. Prompts come in myriad forms, and some are much more effective than others [15]. A prompt can be as simple as ordering the available information, for example, by placing the most important information first and then sequentially including less and less important bits of information. The idea of this prompt is that the ordering signals the importance of the information to the reader, thus encouraging them to pay more attention to earlier information than later. Another example from more contemporary research is using joint rather than separate evaluation to help people identify information gaps [16]. Having multiple options for simultaneous review prompts people to compare and contrast information. Engineering what options are available for simultaneous review can serve to highlight different information, including missing information when it is withheld.

Yet responding to a prompt and noticing missing information is not enough to ensure that people will make appropriate inferences about its value; people also need to consider the correlation structure of the present and missing information. To demonstrate both the relevance of correlation structure, as well as people’s failure to take it into account appropriately, Simmons and Lynch [17] asked study participants to rate the attractiveness of multiple refrigerators in side-by-side comparisons based on either one attribute (capacity: cubic footage) or two attributes (cubic footage plus energy use, shelf space, or warranty). The researchers chose these attributes so that one of them (energy) would be negatively correlated with capacity, a second (shelf space) would be positively correlated, and the third (warranty) would not be correlated. They found that when information was missing, people did not draw the appropriate inferences from the available information—e.g., participants did not use larger capacity to infer more shelf space.

Since this early work, the internet has significantly changed the landscape of consumer choice. New digital media allow businesses unprecedented control over the content people digest—something that Kivetz and Simonson [5] noted nearly 20 years ago. Yet, control over content does not necessarily mean control over the inferences a person will make, including inferences about missing information. Consider, for example, a study by Naylor, Lamberton, and Norton [18] in which the missing information was the identity of a person who had left an online review of a product. Internet retailers choose how much information to disclose about reviewers: Are they in a person’s same demographic? Do they share similar preferences? Was the reviewer given an incentive to provide the review? Given limited information about an anonymous reviewer, it is unclear how a person will infer the reviewer’s tastes, attitudes, and other traits. Naylor, Lamberton, and Norton’s research suggests that an unprompted person will typically infer, egocentrically, that an anonymous reviewer is similar to themselves. They show, however, that priming thoughts about others, and signaling the potential for reviewer heterogeneity, can moderate this effect.

An alternative account of how people handle cases of withheld information is offered by the Persuasion Knowledge Model (PKM), which posits that, over time, people accrue knowledge which helps them cope with attempts at persuasion made by salespeople or delivered in marketing content [1]. According to the PKM, a central tenet of a person’s persuasion knowledge is their perception of tactics or strategies used by agents in these attempts. When a person recognizes that an agent is using a tactic, PKM predicts that the content of the persuasive attempt will take on new meaning, and possibly be discounted. For example, without persuasion knowledge related to the decision to omit information, the omission of a sanitary inspection grade for a restaurant on Yelp may be encoded as a simple oversight or a digital glitch. With persuasion knowledge about why an agent may omit information, according to the PKM, the meaning of the omission changes, and it may be encoded as a nefarious act, even without an explicit prompt.

An important implication of the PKM is that seemingly trivial changes in the structure and format, or mode, of the persuasive attempt, such as—the focus of our investigation—changing from traditional to digital media, can have surprisingly dramatic effects. Thus, all else being equal, making a dining decision using Yelp may recruit different persuasion knowledge than making the same decision based on conversational interaction.

Friestad and Wright [1] point out that persuasion knowledge does more than help consumers avoid being fooled; it guides a person’s attention to critical details, highlights background conditions that might give rise to tactical and strategic choices of a business, and facilitates inferential thought. These processes function to activate the correct coping mechanism for a persuasion attempt. Examples of such mechanisms include openness to information when the model suggests that a business is trying to aid a person in achieving her goals [19] but skepticism when the model suggests that a business is placing its goals before the person’s [20].

### Memory for conversational information

Research suggests that information is handled differently depending on the perceptual processing pathway. A classic experimental setup demonstrating this well-established phenomenon is to vary the information pathway, either visual or auditory, and then measure participants’ recall of the communicated information. This research shows that, for example, study participants who received numbers via an auditory stimulus recalled longer strings of digits than those who received the same information via a visual stimulus [21].

A general finding of the extensive research in this domain is that auditory stimuli tend to lead to higher recall rates than visual stimuli, possibly due to the way that the brain handles information inputs from the different pathways [22, 23]. Audition can also outperform the tactile modality in a pattern recognition task. Participants who heard patterned tones were more likely to correctly identify that two sequences matched than participants who felt patterned vibrations via their fingertips [24].

### Inferences in conversational contexts

Recall that economic theory predicts that people will respond to missing information with extreme skepticism, but considerable empirical research challenges this prediction. The behavior we predict—more negative responses to information omitted in conversational than traditional disclosures—fits better with the PKM prediction. PKM suggests that this behavioral change occurs because the conversational disclosure activates a different schema that catalyzes skepticism to cope with the persuasion attempt. This insight is related to a fundamental idea from the language sciences: In conversational settings, a person will use the content of a message to infer meaning that the sender did not explicitly state [25]. For example, a less informed party may infer that a better-informed party withholds decision-critical information when he has something to hide.

There is considerable evidence in the language sciences and psychology that during conversations people routinely engage in sophisticated reasoning to draw inferences about interlocutors’ character and intentions without any explicit prompting. Hearing a person speak rather than reading their words, for example, can result in higher evaluations of the person’s intellect [26] and may even reduce the propensity to unfairly criticize opponents [27], provided that pauses and disfluencies like uh or um do not fill the speech [28–31]. Moreover, conversational exchanges engage a rich repertoire of skills that are routinely activated in social interactions, including the ability to make situational inferences [32] and think counterfactually about outcomes [33], skills that are essential to understanding why another party might choose to omit information. In negotiations, social interactions can cause buyers to be more cynical towards sellers than they would have been otherwise [34]. Finally, negotiators who adopt a firm communication style receive more desirable counteroffers, thus achieving better outcomes than their warmer counterparts. This outcome is likely a result of inferences based on the negotiator’s style that suggest what ultimate intentions they have [35].

A key idea from language science is that people make pragmatic inferences about the meaning of what is said in a conversation. More than a general inference, a pragmatic inference occurs when a listener uses the choices made by an interlocutor, such as what content she includes or excludes, as well as the form and structure of what she says, to infer meaning that is not explicitly communicated [25]. The principles of conversational pragmatics state that any particular communicative message will be judged in light of other messages that might have been sent and under the assumption that the speaker is trying to be as cooperative as possible [25, 36, 37]. Research suggests that listeners are exquisitely tuned to the resulting nuances. For example, “no rats were seen in the kitchen on three days last week” is typically interpreted as indicating that rats were seen in the kitchen on at least some of the other days, and possibly in other parts of the building even on those three days.

Now consider the Sanitary Inspection Grade (SIG) information available on Yelp about a restaurant being embedded in a simple conversation. If, in response to an inquiry about a restaurant’s SIG, a consumer read or heard, “I don’t know what it is,” “The owner has not added that to our database,” or worse, “The owner has asked that I don’t share that information,” it seems clear what inference she will make: The restaurant has something to hide. In contrast to the rather abstract tabular mode of communication common to digital settings, which has dominated prior research examining the impact of information disclosure, conversational technology potentially engages the powerful reasoning mechanisms inherent in language comprehension.

### Conversational technology

In their provocative survey of machine learning and the future of the service industry workforce, Brynjolfsson and Mitchell [38] posit that chatbots and similar conversational agents are poised to replace many human laborers. Brynjolfsson and Mitchell claim that this is an artifact of how chatbots are trained relative to humans. The machine learning algorithms that serve as the brains of chatbots are trained on vast interactions, more than any single person could ever hope to experience or learn from job training. This training advantage allows chatbots to readily identify which responses to common questions are most likely, for example, to result in sales, and they are thus more likely to say the right thing at the right time to generate sales than their human counterparts. Indeed, recent research demonstrates that undisclosed chatbots outperform their human counterparts in closing sales [39]. Large language models, such as OpenAI’s Generative Pre-trained Transform models (e.g., GPT4, ChatGPT) [40], are accelerating the development of such conversational technologies with their ability to supply near-human responses to queries.

However, technologies do not necessarily need state-of-the-art machine learning capabilities to elicit responses typically reserved for human social interactions. One of the most famous case studies of natural language between man and machine is ELIZA, a technology developed by Joseph Weizenbaum [41] to study conversational human-computer interactions. The ELIZA program included multiple different scripts, including DOCTOR. When executing DOCTOR, ELIZA parodied Rogerian psychotherapy by parroting back a person’s statement as a question. Much to Weizenbaum’s surprise, many of ELIZA’s users reported emotional interactions and ascribed a theory of mind to the machine. In other words, users anthropomorphized the machine and responded as if interacting with an actual human. In computer science, this is known as the ELIZA effect [42]. Importantly, the more human features a machine has, the more likely it is to be anthropomorphized [43]. In the case of speech, Schroeder and Epley demonstrate that simple linguistic cues, like a varied pace in speech, are correlated with an increased likelihood of anthropomorphizing a machine [44]. Recent research has also demonstrated that the ELIZA effect can produce feelings of warmth towards robots [45].

From ELIZA to GPT4, conversational technologies, we argue, recruit a fundamentally different set of cognitive resources than typical print or visual content, just like reading somebody’s words can lead to different evaluations than hearing them. This is supported by research in psychology (e.g., [22, 26, 33]) and the language sciences (e.g., [25, 36, 37]). We hypothesized that recruiting different cognitive resources would alter how information was evaluated, ultimately impacting downstream judgments and decisions. We report three experiments that compare participants’ responses to common, tabular disclosures (currently the most common mode of information disclosure) with their responses to novel disclosure modes involving recorded conversations, unfolding written chats, or transcribed conversations between an informationally advantaged and disadvantaged party (or between a consumer and a conversational technology). We show that these modes cause people to respond to missing information in a manner more reflective of skepticism about withheld information and to do so without an explicit prompt. We provide evidence that conversational technology leads to greater inferences about the persuasion intentions of an agent—inferences that are not made in response to the same information content delivered in a standard format—and to the increased recall of (strategic) omissions.

## Experiments

We adapted the paradigm for the first two studies from Gurney and Loewenstein’s between-subject experiments [16]. Participants in these survey-based experiments were asked to imagine choosing where to go out for dinner with friends, to review a restaurant option, and then indicate their interest in dining at that restaurant. The studies relied on modified screenshots from Yelp in which the sanitary inspection grade (SIG) disclosure varied across conditions while the remaining information stayed constant. Figures are not included due to copyright issues, however they are available from the first author. Experiment 1, below, establishes the effect of getting information in a conversational format by pitting these screenshots against recorded conversations between a patron and a restaurant host. Experiment 2 builds on Experiment 1 by replacing the restaurant host with the Google Assistant in the recorded conversations. In their prior paper, Gurney and Loewenstein demonstrated that people do not assume the worst from missing information; thus, we needed conditions with the various SIG levels (e.g., A, B.) to establish benchmarks against which to measure the conversational stimuli. Experiment 2 also adds unfolding chats and conversation scripts to control for potential processing channel effects, as there is documentation of differential processing for written and spoken dialogue [46, 47]. Finally, Experiment 3 introduces a new choice scenario: hiring a service professional (a dog-walker), demonstrating the effect in a different context. It also takes special care to hold all conditions constant except for the mode of information delivery (conversational versus tabular disclosure).

### Ethics statement

The Carnegie Mellon University Institutional Review Board approved all studies in this paper, and we obtained written informed consent from all participants. We obtained informed consent via a standard Carnegie Mellon University Institutional Review Board online consent form in which participants read information about the study and use radio buttons to confirm that they are at least 18 years old, understand what they read in the consent form, and want to participate in the study.

### Experiment 1: Restaurant sanitary inspection grade (SIG) information in conversations and yelp

#### Method

Participants were asked to imagine being on a trip to Los Angeles with friends and trying to find a restaurant for dinner. They were informed that the group was voting on restaurants as they arose in a search. Each participant saw one of the stimuli described below, and indicated to their friends their endorsement of visiting that restaurant using a slider with responses ranging between 0 (Definitely not) to 100 (Enthusiastic). Then, with the stimulus still displayed on the screen, participants explained the reasoning behind their evaluation of the restaurant in an open response. Later, we coded participants’ responses by indicating whether they spontaneously mentioned the SIG. Participants then progressed to a new page without the restaurant information and answered recall questions about the restaurant. One question checked participants’ recall of the SIG (or its absence). Other questions checked participants’ recall of the remaining information.

**Fig 1:**
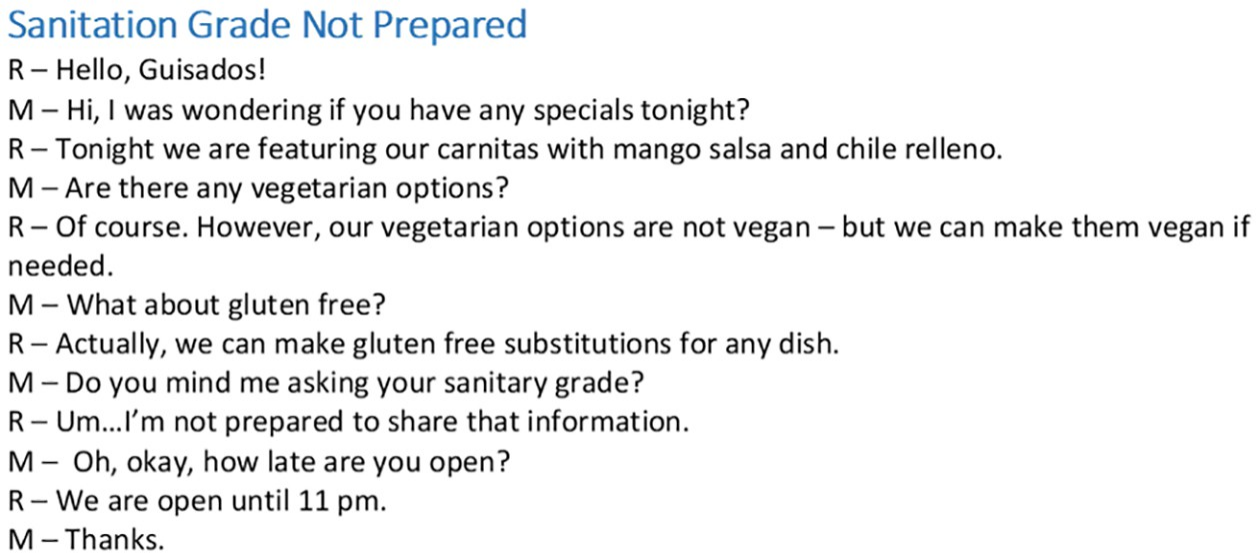
Experiment 1 conversational stimulus example. A representative script from Experiment 1 with the “I’m not prepared to share that information” [INPS] SIG disclosure. “R” is the restaurant and “M” is the potential customer.

Experiment 1 included 17 different conditions. Five modified Yelp screenshots were used as the standard information mode controls (SIG disclosures: A, B, C, a dash to indicate that the SIG was missing, and “Not Reported by Owner” [NRBO]). We contrasted these conditions with six recorded conversation conditions (Fig 1 is an example script; SIG disclosures: A, B, C, “We don’t have a sanitary inspection grade” [DNH], “I’m not prepared to share that information” [INPS], and “[pause] I’m not prepared to share that information” [INPSp]). The SIG is the only information that varied within the information presentation modes. The Yelp screenshots and recordings had much of the same content, but, because some of the information in the Yelp screenshots was not easily operationalized in the conversations, there were some limited differences in the content across the two information presentation modes (we remedy this in the following studies). Therefore, we also ran six conditions in which participants interacted with both spoken and visual stimuli to ensure full information access. Table 1 summarizes these conditions.

**Table 1:** Experiment 1 treatment conditions.

Yelp	Phone Conversation	Yelp & Conversation
A	A	A
B	B	B
C	C	C
Dash, i.e., “-”	“We don’t have a sanitation inspection grade”	Dash & “We don’t have a sanitation inspection grade”
Not Reported by Owner	“I’m not prepared to share that information”	Dash & “I’m not prepared to share that information”
	[pause] “I’m not prepared to share that information”	Dash & [pause] “I’m not prepared to share that information”

The 17 treatment conditions of Experiment 1. The cells describe the SIG disclosure(s), the only information that varied across conditions, seen by participants in each condition. The following two studies improve on the information consistency across the treatment conditions; Study 3 ensures that the exact same information was made available.

#### Participants

One thousand eight hundred eleven participants were recruited using Amazon Mechanical Turk to complete a survey-based experiment on the Qualtrics platform (approximately 100 per condition; because of randomization, there was a slight variation in the number of participants assigned to each). Data collection began on 6 June, 2017. Forty-nine percent of participants were male (n = 894), the average age of a participant was 35.5 years (min = 18, max = 88), 54% had a bachelor’s degree or higher (n = 974), and the median household income range was $25,000–$50,000 (n = 519).

#### Results

**Fig 2:**
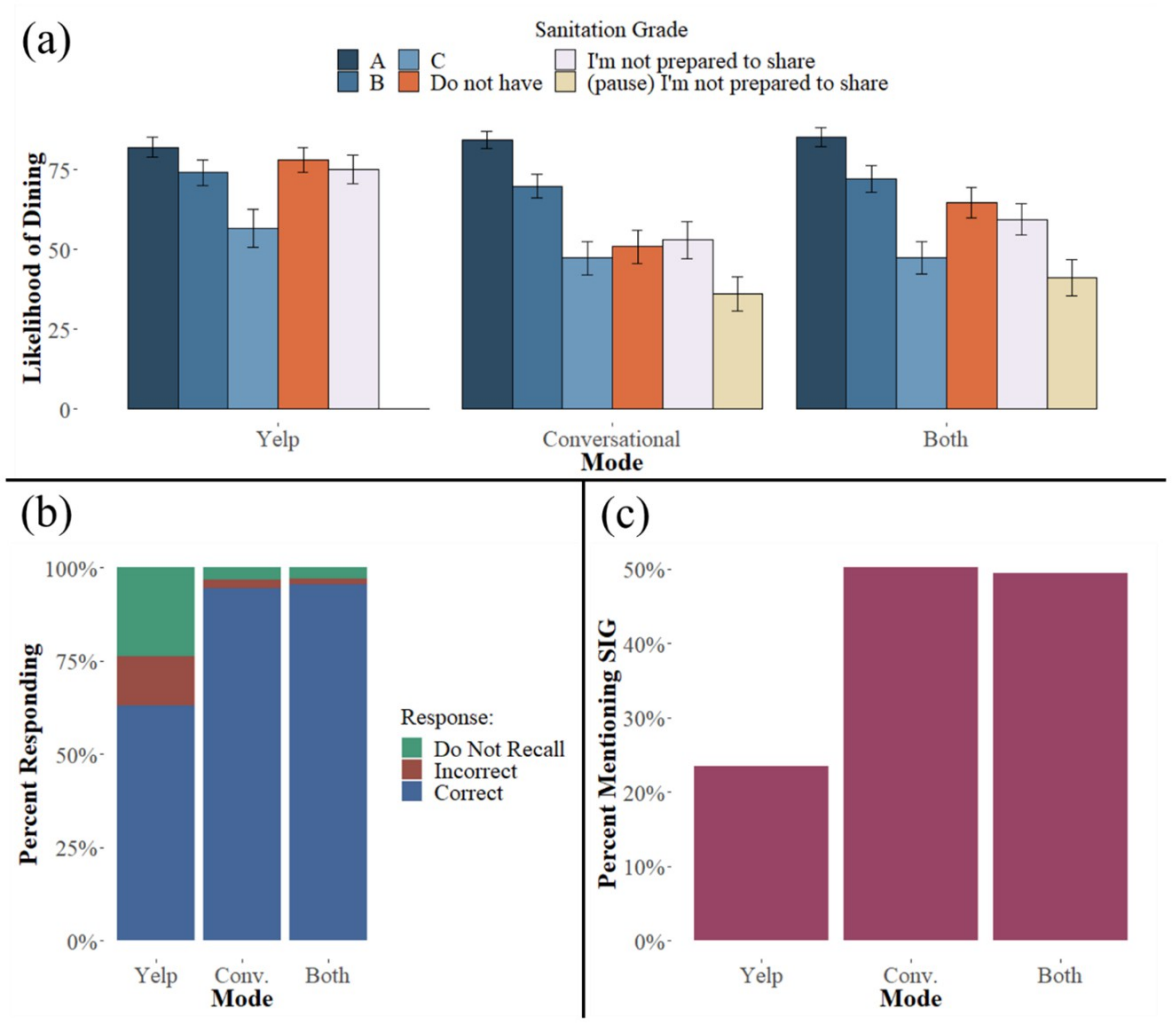
Results from Experiment 1. Panel (a) presents the outcome (averages with 95% CI bars) for the main dependent variable of experiment 1, reported endorsement of dining at the restaurant on a scale of 0 (definitely not) to 100 (enthusiastic!), for each of the 17 treatment conditions. The data are grouped by the three disclosure modalities: Yelp screenshot, spoken (recorded phone conversation), and both. The disclosure conditions are letter grades of A, B, and C and nondisclosures of “We don’t have a sanitary inspection grade” (DNH), “I’m not prepared to share that information” (INPS), and “(pause) I’m not prepared to share that information” (INPSp). Panel (b) presents the percentages of participants in each category who recalled, did not recall, or incorrectly recalled the SIG. Panel (c) shows the percentage of participants in each category who cited the restaurant’s sanitation as being influential in their judgment about dining there.

Fig 2 shows the results of the experiment graphically, and S1–S3 Tables present regression analyses of our main predictions, examining first the impact of the absence of the SIG under the different modes, second the effect of time delay in the conversational mode conditions, and third the effect of information presentation mode on participants’ responsiveness to (non-missing) SIG grades (A, B, and C), respectively. All the regression specifications include a dummy variable for the presence of conversational mode and a second dummy for the situation in which both the Yelp and the conversational modes were presented. Interactions between these indicator variables and relevant independent variables are also included (the presence/absence of SIG information, the presence/absence of time delay, and a variable representing letter grade, coded as 4, 3, and 2, as often done in educational settings). Two versions of each regression are always presented, the second with demographic controls for age, gender, education, and income. This same basic structure is repeated for experiments 2 and 3.

As evident in Fig 2a, the conversational mode leads to a much stronger, negative response to the absence of information (S1 Table presents analyses of just the missing information conditions). Putting disclosure of missing information in a conversational mode dramatically reduced willingness to dine at the restaurant when SIG information was missing. Although we thought that pairing the standard Yelp disclosure mode with the conversational mode would have a similar effect to the conversational mode alone, the figure and regressions yielded an interesting pattern: Providing the Yelp-style mode reduced the conversational mode’s effectiveness in producing skepticism toward a missing SIG.

Regression analyses of the delayed response conditions demonstrate that conversational modes can provide additional dimensions of information that are not easily communicated in tabular modes (S2 Table presents analyses of just the conditions with a pause before INPS). Hesitation on the part of the restaurant receptionist before withholding the SIG resulted in significantly lower ratings of willingness to dine at the target restaurant. For the delayed response, the effect of pairing the standard and conversational disclosure was not significantly different from just the conversational disclosure alone but was directionally consistent with the possibility that pairing the two modes reduces the effectiveness of the conversational format.

Restricting the sample to those who received letter grade SIGs (S3 Table presents analyses of just the conditions with a letter grade) revealed an interaction between the conversational modes and grades, such that the conversational modes (as well as the combination of conversational and standard modes), relative to the standard format, led to participant responses that were higher when the grade was an A and lower when the grade was a C. Conversational modes, therefore, not only made respondents more sensitive to the absence of information but also more sensitive to the specific value of the SIG when it was present. Although not in our hypothesized effects, this outcome is consistent with the PKM’s assertion that information presentation modes can have broad impacts on how people make sense of and use information.

Prior research indicates that a conversational mode of information delivery may influence short-term memory for communicated information [22]. The second set of analyses examined the impact of the two different modes of disclosure (and their combination) on the recall of SIG information rather than an interest in dining at the restaurant. In addition to predicting the desire to dine at the restaurant, the treatment condition also predicted the likelihood that a participant recalled the value, or presence, of the SIG disclosure (Fig 2b). Whether a grade was present Column 3), a conversational mode led to a significantly higher likelihood of correct recall of the (non) disclosed information, providing further evidence that the conversational mode influenced the impact of the information.

Lastly, we examined the conversational mode’s impact on the degree to which participants reported using the SIG information (coded as 1 whenever the SIG was mentioned) in deciding on their likelihood of dining (Fig 2c). Participants were significantly more likely to mention the SIG when it was withheld in a conversational stimulus than when it was withheld in a Yelp stimulus (S1 Table Column 5). Those participants who listened to a conversation with the pause were even more likely to mention the SIG in their open response (S2 Table Column 5). A chi-squared test was performed to examine the relationship between grade value, good (A) or bad (C), and the inclusion of SIG in an open response. Participants who interacted with a C stimulus were significantly more likely to mention the SIG in their open response (*χ*^2^(1, *n* = 631) = 67.58, *p* < .001), verifying that the information is considered when present.

We undertook Experiment 1 to establish the basic effects of conversational disclosures, relative to more standard tabular ones, on the use of information during judgments. The conversational modes of this study produced much stronger, negative responses to the absence of information and a higher likelihood of recalling that it was missing. Interestingly, when the two modes were paired, the conversational effect was weaker. We also demonstrated that the conversational mode affords additional dimensions of information that are not readily communicated in tabular modes. Specifically, a delayed response resulted in significantly lower evaluations of the target restaurant. Study participants who interacted with the conversational mode were also more likely to report using the critical information in their judgments. These results motivated us to design Experiment 2, in which we translate from a human-human conversational stimulus to a human-machine conversational stimulus.

### Experiment 2: Conversational disclosures and the google assistant

Experiment 1 demonstrates that a conversational mode can influence choice outcomes. It did not, however, explicitly test the effect of a conversational technology, such as the Google Assistant. It seems reasonable that a participant may draw different conclusions if they believe that the information source is a human versus a conversational technology (e.g., they could weigh the information differently when it comes from a human rather than a machine). Thus, in order to better identify the effect of conversational technology relative to standard modes of information disclosure on choices involving missing information, we implemented two Google Assistant (GA) modes in Experiment 2. In one, participants read the script of an interaction between a potential restaurant patron and the Google Assistant, and in the other they listened to a recording of the same interaction.

#### Method

**Fig 3:**
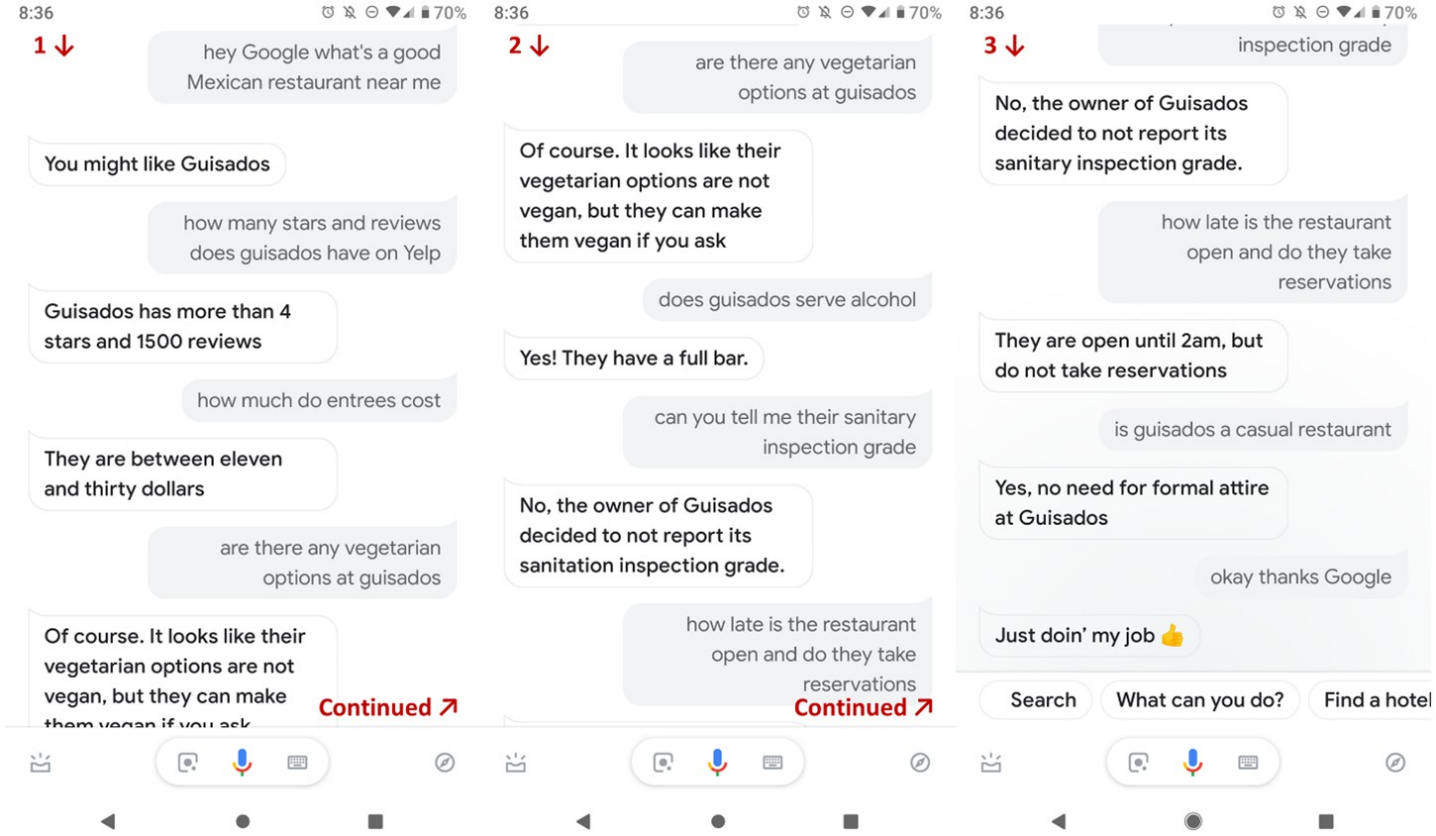
Chat stimulus script from Experiment 2. A representative chat from Experiment 2. The Google Assistant responses are white and the patron’s are gray.

Participants were given the same basic scenario as in Experiment 1. Those who were in the standard information format conditions were given Yelp screenshots as in Experiment 1. Participants in the conversational technology conditions were told that their friend used the Google Assistant, rather than Yelp, to find a restaurant. These participants saw screenshots of the hypothetical chats with the GA or heard a recording of the same spoken conversations (see Fig 3). The conversations were programmed into the GA so that it responded with the necessary information. Note that there were some minor differences between these and those of Experiment 1 because of how a person must interact with the GA.

Since they do not bear directly on our hypotheses, we did not include the C, pause, and all unfolding chat conditions. This left us with a three (Yelp, recorded conversation with the GA, screenshots of the conversation with the GA) by three (A, B, NRBO) experiment. Table 2 summarizes the conditions. We also transitioned to a scale anchored at “Definitely Not” and “Definitely” rather than “Definitely Not” and “Enthusiastic.”

**Table 2:** Experiment 2 treatment conditions.

Yelp	Google Assistant Conversation—Voice	Google Assistant Conversation—Chat
A	A	A
B	B	B
Not Reported by Owner	“The owner of Guisados decided to not report its [SIG]”	“The owner of Guisados decided to not report its [SIG]”

9 treatment conditions of Experiment 2. The cells describe the SIG disclosure(s), the only information that varied across conditions, seen by participants in each condition.

#### Participants

Nine hundred and five participants were recruited using Amazon Mechanical Turk to complete a survey-based experiment on the Qualtrics platform (approximately 100 per condition; because of randomization, there was a slight variation in the number of participants assigned to each condition). Data collection began on 14 March, 2019. Fifty-one percent of the participants were male (n = 458), the average age of a participant was 37.68 years (min = 18, max = 74), 51% of them had a bachelor’s degree or higher (n = 463), and the median household income range $25,000—$50,000 (n = 263).

#### Results

**Fig 4:**
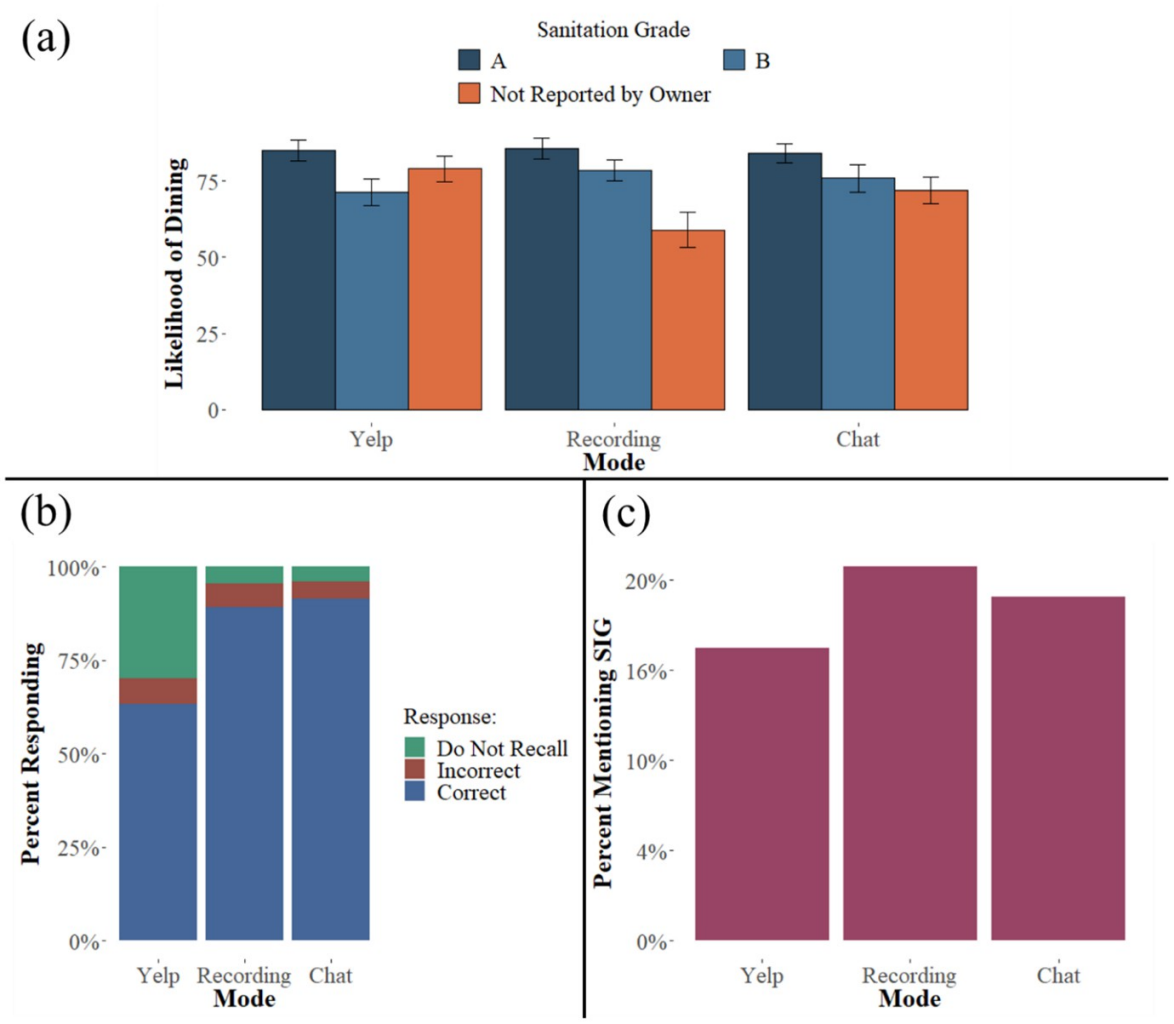
Results from Experiment 2. Panel (a) presents the outcome (averages with 95% CI bars) for the main dependent variable of experiment 2, reported endorsement of dining at the restaurant on a scale of 0 (definitely not) to 100 (definitely), for each of the 9 treatment conditions. The data are grouped by the three modes of disclosure: Yelp screenshot, spoken (the recorded Google Assistant conversations), and chat (screenshots of the Google Assistant conversations). The information conditions are letter grades of A and B, plus the nondisclosures, which were the phrase “Not Reported by Owner” in visual condition and the statement “the owner would rather not disclose that information” in the recording and chat conditions. Panel (b) presents the percentages of participants in each category who recalled, did not recall, or incorrectly recalled the SIG. Panel (c) shows the percentage of participants in each category who cited the restaurant’s sanitation as being influential in their judgment about dining there.

Fig 4 presents the results of Experiment 2 graphically, and S4 and S5 Tables present regression analyses of our main predictions. S4 Table examines the impact of the absence of the SIG under the different modes of information presentation, and S5 Table shows the effect of information presentation mode on participants’ responsiveness to SIGs. Each regression specification includes dummy variables for the conversational modes. Interactions between these indicator variables and relevant independent variables were also included (grades coded as in Experiment 1).

The results of Experiment 2 further support our hypotheses, including that the conversational effect observed in Experiment 1 replicates when the interlocutor is a conversational technology. Operationalizing disclosure as a conversation with the GA, whether participants listened to a recording or saw screenshots of the chat, caused a significant reduction in willingness to dine at the restaurant when the SIG was missing (Fig 4a; S4 Table Column 1). The impact of the two types of conversational disclosure (chat screenshots and spoken) on recall of missing SIG information echoed the results of Experiment 1. When the information was missing, participants who encountered a conversational disclosure were much more likely to correctly recall that it was missing (Fig 4b; S4 Table Column 3).

The effect did not hold for disclosed SIGs, a different outcome than observed in Experiment 1. Analyzing the sample that reviewed a stimulus with a letter SIG did not follow the results of Experiment 1. It should be noted, however, that the range of grades in this experiment (A and B) was smaller than the range of grades in the prior studies (A, B and C).

As in Experiment 1, we investigated the impact of conversational mode on participants’ reports that they used the SIG information in deciding on their likelihood of dining at the restaurant (Fig 4c). When the SIG information was withheld, participants who interacted with the chat screenshot and spoken stimuli were significantly more likely to mention the SIG in an open response to the question about their judgment of the restaurant (S4 Table Column 5). When the SIG was present as a letter grade, unlike in the prior studies, there was not a higher likelihood of mentioning it. In sum, Experiment 2 supported our prediction that the conversational mode used by technology like the Google Assistant elicits different responses than other modes of conveying information, including better recall of information being withheld and an increased likelihood of inferential thinking about withheld information.

As noted, both the recording of a conversation with the GA and the chat images resulted in significantly more skepticism toward the missing SIG relative to the Yelp screenshot. The difference between the two conversational stimuli was also significantly different, albeit to a lesser extent (the recording was more effective than the chat). The difference between the chat and recorded stimuli observed in Experiment 2 may reflect information introduced by the non-verbal aspects of spoken dialogue.

### Experiment 3: Generalization of conversational disclosures in technology and refining experimental controls

Experiment 2 shows that the effects of conversational disclosure extend to dialogues with conversational technologies. It and the previous study, however, examined the same dining-out scenario. Additionally, the operationalization of the withheld information varied across media, and the information source was not held perfectly constant. Experiment 3 shows the generality of the effect in a new choice setting—specifically, hiring a service professional—and better controls for source effects and how the withholding of information is operationalized.

#### Method

Experiment 3 implemented a simple, preregistered two-by-two survey-based design: standard mode (screenshot) or conversational (recorded Google Assistant conversation) crossed with information available (a customer reference) or not (a statement saying the service provider had not enabled the customer references feature), all of which are summarized in Table 3. Participants were asked to imagine that they had been taking their dog to a daycare facility and noticed that the dog seemed depressed at the end of each day—potentially a result of mistreatment. Rather than looking for another daycare provider, they decide to leave the dog at home during the day and hire a dog walker who has passed a background check to stop by in the evenings when they must work late. Participants were further informed that they are considering a dog walker found using a hypothetical Google service called Google Pros, a directory for local professionals. Participants in the screenshot conditions saw an image designed to resemble a screenshot of a mobile device display. Those in the conversational conditions listened to a recorded conversation with the GA. Participants in the available information conditions saw or heard the same customer reference, “[The service provider] is nice, and the dog seems to like him. However, he is occasionally late.” Likewise, participants in both unavailable information conditions learned: [The service provider] has not enabled the customer references feature on his account. All other information was held constant across the two different formats. Example stimuli are available from the first author as copyright issues prevented their publication.

**Table 3:** Experiment 3 treatment conditions.

Google Pros Screenshot	Google Assistant Conversation—Voice
“[The person] is nice, and the dog seems to like him. However, he is occasionally late.”	“[The person] is nice, and the dog seems to like him. However, he is occasionally late.”
“[The person] has not enabled the customer references feature on his account.”	“[The person] has not enabled the customer references feature on his account.”

4 treatment conditions of Experiment 3. The cells include the customer references statement seen or heard by participants, the only information that varied across conditions.

As in the previous studies, participants had access to their assigned stimulus while completing the main dependent measure, which was the likelihood that they would hire the dog walker in the stimulus. This likelihood was elicited using a slider [-100, 100] with seven labels ranging from extremely unlikely to extremely likely. The two visual stimuli were designed and pretested to ensure that they elicited similar likelihood ratings. Participants were also asked to explain the reasoning behind their responses in an open-response question. After completing the main dependent measures, participants advanced to a new page and answered secondary questions about the content of the stimulus they interacted with.

Our prediction is that participants who encounter missing information in the GA-based stimulus will report a lower likelihood of hiring the service professional, be more likely to recall that a reference was not available, and be more likely to mention it in their open response about why they made the choice that they did.

#### Participants

Eight hundred and one participants were recruited using Amazon Mechanical Turk to complete a survey-based experiment on the Qualtrics platform (approximately 200 participants assigned to each condition). Data collection began on 29 September, 2019. Forty-two percent of the participants were male (n = 335), the average age of a participant was 39.78 years (min = 8, max = 81), 54% of them had a bachelor’s degree or higher (n = 434), and the median household income range was $50,000—$75,000 (n = 200).

#### Results

**Fig 5:**
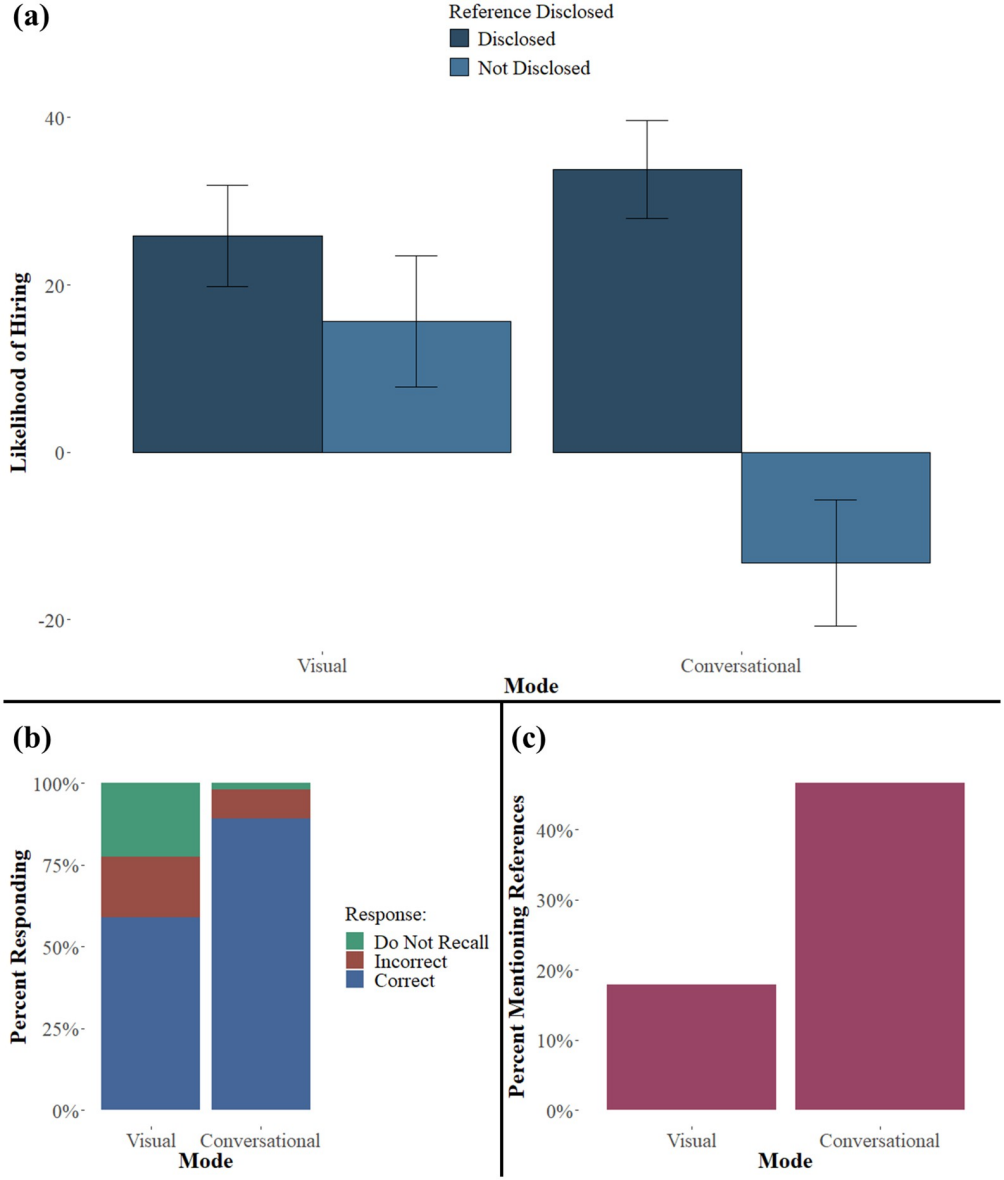
Results from Experiment 3. Panel (a) presents the outcome (averages with 95% CI bars) for the main dependent variable of Experiment 3, likelihood of hiring the service provider on a scale of -100 (extremely unlikely) to 100 (extremely likely), for each of the 4 treatment conditions. The data are grouped by the disclosure modes: Google Assistant (Conversational) and Google Screenshot (Visual). Panel (b) presents the percentages of participants in each category who recalled, did not recall, or incorrectly recalled the availability of the reference feature. Panel (c) shows the percentage of participants in each category who cited the reference feature as being influential in their judgment about hiring the service provider.

Fig 5 presents the results of Experiment 3 graphically; S6 Table presents regression analyses of our main predictions. Each regression specification includes indicator variables for a conversational technology stimulus, whether the information was withheld, and the interaction of these indicators. As with previous tables, both a basic regression and one that includes demographic controls are reported for each dependent measure.

The results of Experiment 3 add validity to our hypotheses. Participants who reviewed the GA stimulus in which the customer reference feature was “not enabled” reported a significantly lower likelihood of hiring the service provider (Fig 5a, S6 Table Column 1). Participants who listened to the GA conversation, relative to those who saw the screenshot, were much more likely to correctly recall its availability (Fig 5b; S6 Table Column 3). To interpret the difference in difference, we sum the four coefficients of the model to compute the expected change in the log odds of recalling the availability of the customer references feature, which also suggests an increased likelihood of recall (*β*_0_+ *β*_*conversational*_ + *β*_*withheld*_ + *β*_*conversational*:*withheld*_ = 1.566). Participants who interacted with one of the GA stimuli were also more likely to mention the term “reference” in the open response question than participants who interacted with a screenshot stimulus (Fig 5c; S6 Table Column 5. There was also a main effect for withheld, but not an interaction effect.

Experiment 3 thus adds credence to the results of the previous studies. Beyond generalizing the effects of information (non) disclosure via a conversational technology to a domain different from Experiments 1 and Experiment 2, the informational content is especially carefully controlled by incorporating exact comparability between the conversational and non-conversational conditions information source (the same source was cited in all conditions) and the stated reason for the missing information (the same nondisclosure statement was conveyed in the visual and conversational stimuli).

## Discussion

In three experiments, we demonstrate that conversation, specifically conversational technology (Experiment 2 and Experiment 3), elicits different responses to missing information than other communication media. In all three studies, participants who received information in a conversational mode instead of a standard mode reported a significantly lower likelihood of using a service when information was (strategically) omitted. In open-ended responses about their reasoning, in our restaurant scenarios, participants who received information in a conversational mode were more likely to cite the missing sanitation information as a primary reason for their decision, which we interpret as evidence that they engaged persuasion knowledge and considered the pragmatic implicature of an omission. Experiment 2 validated the results of Experiment 1 with stimuli designed to mimic the actual use of conversational technology. Participants who interacted with a stimulus created using the Google Assistant, on average, reported a significantly lower likelihood of dining at the target restaurant when information was missing than participants who interacted with a Yelp stimulus that conveyed the same information. Experiment 3 generalized the results of Experiments 1 and 2 by introducing a new choice setting, better controlled for the way information was withheld and disclosed, and relied on one information source. In all studies, participants who interacted with a conversational mode were significantly more likely to remember that information was omitted than their counterparts who interacted with standard modes.

These results are significant in at least three ways. First, they suggest that findings from earlier studies that found insensitivity to omitted information may not generalize to conversational technology. Second, they suggest that firms must take seriously the pragmatic and semantic content they communicate using conversational technology. Third, they point to the potential for fruitful future theoretical and empirical interaction between consumer behavior, economics, and the language sciences. More precisely, our results fit with the observation from conversational pragmatics that the interpretation of conversational input is tuned to what is said and what might have been said but was not. The PKM predicts that this will be the case in consumer settings and that the information mode will impact consumers’ ability to apply their knowledge of persuasion tactics. Our results support this prediction and provide new insights into the applicability of the economic models. It seems that consumers can make the inferences predicted by the models of unraveling theory, provided that the information is presented in a manner that elicits their rational capacities.

### Limitations

The studies that we report are hypothetical. Some research posits that hypothetical choice experiments are vulnerable to bias [48, 49]. A meta-analysis of economic evaluations, however, shows that evaluations in hypothetical experiments do not meaningfully differ from the outcomes of real-choice experiments [50]. Additionally, Camerer and Hogarth [51] reviewed 74 experiments and concluded that there was no evidence that hypothetical scenarios resulted in more biased decision making than studies involving real choices.

### Future directions

Widespread adoption of conversational technology has allowed us to ask questions about how people interact with intelligent conversational agents like Siri, Alexa, and the Google Assistant. A decade ago, since few people had access to them, asking how such agents influence decisions was only hypothetical. These agents are now available to the majority of people at any moment. The hypotheses tested herein seek to contribute to an emerging field of research that examines diverse dimensions of consumer responses to artificial agents.

We study a narrow subfield of behavioral responses to information: how people respond to omissions of decision-critical information. Omissions are not, however, the only variety of malicious information provision. It is not uncommon, for example, to see a better-informed party attempting to distract a less-informed party from pertinent information—including an omission—by providing extra, spurious information. Drug labels, for example, may dilute really significant safety warnings (e.g., that a product could cause heart attacks) with warnings of innocuous consequences (e.g., that the product could lead to dry mouth). Maybe there is a perverse effect of conversational disclosures relative to tabular formats when an agent provides an *excess* of information. The differences we document herein are likely only a few examples of many differences between conversational and standard disclosure formats that warrant investigation.

The open-response questions we asked participants serve to shed light on the psychology, and specifically inferences, driving their responses to conversational information. It is reasonable to believe that a rich set of cognitive processes is employed to make sense of information disclosures, including conversational ones. Undoubtedly, each format will elicit unique inferences about an agent’s intentions. Further exploration of the inferences derived in both information formats is needed to understand how and predict when people form particular inferences about the intentions of others in conversational technology-mediated decisions.

## Conclusion

Prior research suggests that the inferences people draw about missing information, particularly in consumer settings, are not sensitive to changes in the information presentation format [5, 52]. Our results tell a different story that is more relevant now than ever. Voice-interface virtual assistants are increasingly the platform of choice for business-to-consumer interactions. We argue that the conversational nature of these interactions can result in people using different persuasion knowledge than when they use other media formats. The blurring line between human virtual agents will likely strengthen this effect [44]. An apparent conclusion is that businesses must adapt to what persuasion knowledge consumers are using in these modes. Omitting relevant information, such as how similar a reviewer is to a shopper [18], to curate consumer opinions may no longer be a business’s best option. For now, one can only speculate about how conversing with an artificial agent that uses information from similar consumers’ reviews to construct responses will impact a person. In short, because of the rise of conversational interfaces, content creators need to account for the interaction between the mode in which information is presented and what information is and is not presented—and policymakers need to account for the exploitation of information mode in consumer settings.

## Supporting information

S1 Table The effect of Yelp and conversational withholding of SIG information on judgments. Each column is a different regression model. Standard errors are in parentheses and interactions are indicated by a colon. Regression specifications are:
Willingness to dine response (the intercept is a Yelp screenshot with a dash in the place of the grade) regressed on Conversational (the “Spoken” condition with the statement “We don’t have a sanitation inspection grade”) and Both indicators interacted with Omitted indicator (Not Reported by Owner or “I’m not prepared to share that information.”).Specification (1) plus controls for age, male, education (1 if > = bachelors), and income (>$75k annually).Outcome variable is an indicator for correctly recalling the SIG (1 if true) with same IV’s as (1).Specification (3) with same IV’s as (2).Outcome variable is an indicator for if a participant mentioned SIG in an open response about their decision regressed on same IV’s as (1).Specification (5) with same IV’s as (2).
(PDF)

S2 Table The effect of a speech delay in Yelp and conversational withholding of SIG information on judgments. Each column is a different regression model. Standard errors are in parentheses and interactions are indicated by a colon. Regression specifications are:
Willingness to dine response regressed on Conversational (Spoken) indicators down-selected to include only the delayed response conditions. The intercept is computed from the cases in which participants saw the Yelp screenshot and listened to the stimulus with the delay.Specification (1) plus controls for age, male, education (1 if > = bachelors), and income (>$75k annually).Outcome variable is an indicator for correctly recalling the SIG (1 if true) with same IV’s as (1).Specification (3) with same IV’s as (2).Outcome variable is an indicator for if a participant mentioned SIG in an open response about their decision regressed on same IV’s as (1).Specification (5) with same IV’s as (2).
(PDF)

S3 Table The effect of Yelp and conversational disclosure of SIG scores on judgments. Each column is a different regression model. Standard errors are in parentheses and interactions are indicated by a colon. Regression specifications are:Willingness to dine response regressed on Conversational (Spoken) and Both indicators interacted with linear grades (A = 4, B = 3, C = 2).Specification (1) plus controls for age, male, education (1 if > = bachelors), and income (>$75k annually).Outcome variable is an indicator for correctly recalling the SIG (1 if true) with same IV’s as (1).Specification (3) with same IV’s as (2).Outcome variable is an indicator for if a participant mentioned SIG in an open response about their decision regressed on same IV’s as (1).Specification (5) with same IV’s as (2).
(PDF)

S4 Table The effect of Yelp and google assistant screenshot (Chat) and recorded (Spoken) withholding of SIG information on judgments. Each column is a different regression model. Standard errors are in parentheses and interactions are indicated by a colon. Regression specifications are:
Willingness to dine response regressed on Chat and Spoken indicators.Specification (1) plus controls for age, male, education (1 if > = bachelors), and income (>$75k annually).Outcome variable is an indicator for correctly recalling the SIG (1 if true) with same IV’s as (1).(3) with same IV’s as (2).Outcome variable is an indicator for if a participant mentioned SIG in an open response about their decision regressed on same IV’s as (1).(5) with same IV’s as (2).
(PDF)

S5 Table The effect of Yelp and google assistant (Chat and Spoken) disclosure of SIG scores on judgments. Each column is a different regression model. Standard errors are in parentheses and interactions are indicated by a colon. Regression specifications are:
Willingness to dine response regressed on Chat and Spoken indicators interacted with linear grades (A = 4, B = 3).Specification (1) plus demographic controls for age, male, education (1 if bachelors or higher), and income (>$75k annually).Outcome variable is an indicator for correctly recalling the SIG (1 if true) with same IV’s as (1).(3) with same IV’s as (2).Outcome variable is an indicator for if a participant mentioned SIG in an open response about their decision regressed on same IV’s as (1).(5) with same IV’s as (2).
(PDF)

S6 Table The effect of Google and Google Assistant (Spoken) disclosure of customer reference availability on judgments. Each column is a different regression model. Standard errors are in parentheses and interactions are indicated by a colon. Regression specifications are:
Likelihood of hiring service provider response regressed on interaction of Conversational and Withheld indicators.Specification (1) plus controls for age, male, education (1 if > = bachelors), and income (>$75k annually).Outcome variable is an indicator for correctly recalling the availability of a customer reference (1 if true) with same IV’s as (1).(3) with same IV’s as (2).Outcome variable is an indicator for if a participant mentioned customer references in an open response about their decision regressed on same IV’s as (1).(5) with same IV’s as (2).
(PDF)

S1 Checklist Human participants research checklist. (DOCX)

S1 Data (ZIP)

S2 Data (ZIP)
