# Supplementary material for: Conversational technology and reactions to withheld information
Source: PLoS One. 2024 Apr 11;19(4):e0301382. doi: 10.1371/journal.pone.0301382 (PMC11008880; doi:10.1371/journal.pone.0301382)
Supplement: S1 Table — Each column is a different regression model. Standard errors are in parentheses and interactions are indicated by a colon. Regression specifications are: Willingness to dine response (the intercept is a Yelp screenshot with a dash in the place of the grade) regressed on Conversational (the “Spoken” condition with the statement “We don’t have a sanitation inspection grade”) and Both indicators interacted with Omitted indicator (Not Reported by Owner or “I’m not prepared to share that information.”).Specification (1) plus controls for age, male, education (1 if > = bachelors), and income (>$75k annually).Outcome variable is an indicator for correctly recalling the SIG (1 if true) with same IV’s as (1).Specification (3) with same IV’s as (2).Outcome variable is an indicator for if a participant mentioned SIG in an open response about their decision regressed on same IV’s as (1).Specification (5) with same IV’s as (2). (PDF) [file pone.0301382.s001.pdf]

	What do you think about going to Guisados?		What was Guisados' [SIG]?		Why did [the participant] give Guisados that rating?	
	0 (Definitely not) - 100 (Enthusiastic)		Correctly Recalled		Reason Included SIG	
	OLS		*logistic*		*logistic*	
	(1)	(2)	(3)	(4)	(5)	(6)
Intercept (Yelp)	78.020***	84.501***	0.219	0.213	−2.762***	−2.876***
	(2.565)	(4.595)	(0.200)	(0.495)	(0.421)	(0.540)
Spoken	−27.254***	−26.980***	3.068***	3.074***	3.375***	3.404***
	(3.545)	(3.564)	(0.547)	(0.551)	(0.466)	(0.468)
Both	13.806***	13.810***	−0.483	−0.432	−0.823**	−0.831**
	(3.509)	(3.527)	(0.660)	(0.663)	(0.279)	(0.282)
Score Withheld	−3.048	−2.448	0.378	0.372	1.096*	1.088*
	(3.576)	(3.587)	(0.284)	(0.290)	(0.497)	(0.498)
Spoken:Score Withheld	5.171	4.999	−0.995	−0.977	−1.894***	−1.908***
	(4.993)	(5.000)	(0.702)	(0.705)	(0.569)	(0.571)
Both:Score Withheld	−7.370	−7.561	0.483	0.446	0.898*	0.931*
	(4.962)	(4.987)	(0.861)	(0.865)	(0.391)	(0.394)
Dem. Controls		✓		✓		✓
Observations	640	640	640	640	640	640
R^2	0.138	0.145				
Adjusted R^2	0.131	0.132				
Log Likelihood			−231.080	−228.025	−362.855	−361.782
Akaike Inf. Crit.			474.161	476.050	737.710	743.565
F Statistic	20.306*** (df = 5; 634)	11.831*** (df = 9; 630)				

Note: *p<0.05; **p<0.01; ***p<0.001
