# Supplementary material for: Conversational technology and reactions to withheld information
Source: PLoS One. 2024 Apr 11;19(4):e0301382. doi: 10.1371/journal.pone.0301382 (PMC11008880; doi:10.1371/journal.pone.0301382)
Supplement: S2 Table — Each column is a different regression model. Standard errors are in parentheses and interactions are indicated by a colon. Regression specifications are: Willingness to dine response regressed on Conversational (Spoken) indicators down-selected to include only the delayed response conditions. The intercept is computed from the cases in which participants saw the Yelp screenshot and listened to the stimulus with the delay.Specification (1) plus controls for age, male, education (1 if > = bachelors), and income (>$75k annually).Outcome variable is an indicator for correctly recalling the SIG (1 if true) with same IV’s as (1).Specification (3) with same IV’s as (2).Outcome variable is an indicator for if a participant mentioned SIG in an open response about their decision regressed on same IV’s as (1).Specification (5) with same IV’s as (2). (PDF) [file pone.0301382.s002.pdf]

	What do you think about going to Guisados?		What was Guisados' [SIG]?		Why did [the participant] give Guisados that rating?	
	0 (Definitely not) - 100 (Enthusiastic)		Correctly Recalled		Reason Included SIG	
	OLS		*logistic*		*logistic*	
	(1)	(2)	(3)	(4)	(5)	(6)
Intercept (Both)	59.324***	71.059***	2.669***	2.362*	−0.111	0.148
	(2.776)	(5.691)	(0.391)	(0.957)	(0.193)	(0.423)
Delay	−18.222***	−18.713***	0.356	0.351	1.418***	1.449***
	(3.926)	(3.916)	(0.602)	(0.608)	(0.304)	(0.307)
Spoken	−6.435	−5.931	0.000	−0.066	−0.074	−0.080
	(3.926)	(3.923)	(0.553)	(0.559)	(0.273)	(0.276)
Delay:Spoken	1.494	1.733	0.511	0.584	−0.208	−0.193
	(5.566)	(5.546)	(0.926)	(0.934)	(0.422)	(0.425)
Dem. Controls		✓		✓		✓
Observations	430	430	430	430	430	430
R^2	0.093	0.110				
Adjusted R^2	0.086	0.096				
Log Likelihood			−85.741	−82.844	−266.217	−264.219
Akaike Inf. Crit.			179.482	181.687	540.435	544.439
F Statistic	14.530*** (df = 3; 426)	7.479*** (df = 7; 422)				

Note: *p<0.05; **p<0.01; ***p<0.001
