# Supplementary material for: Conversational technology and reactions to withheld information
Source: PLoS One. 2024 Apr 11;19(4):e0301382. doi: 10.1371/journal.pone.0301382 (PMC11008880; doi:10.1371/journal.pone.0301382)
Supplement: S3 Table — Each column is a different regression model. Standard errors are in parentheses and interactions are indicated by a colon. Regression specifications are:Willingness to dine response regressed on Conversational (Spoken) and Both indicators interacted with linear grades (A = 4, B = 3, C = 2).Specification (1) plus controls for age, male, education (1 if > = bachelors), and income (>$75k annually).Outcome variable is an indicator for correctly recalling the SIG (1 if true) with same IV’s as (1).Specification (3) with same IV’s as (2).Outcome variable is an indicator for if a participant mentioned SIG in an open response about their decision regressed on same IV’s as (1).Specification (5) with same IV’s as (2). (PDF) [file pone.0301382.s003.pdf]

	What do you think about going to Guisados?		What was Guisados' [SIG]?		Why did [the participant] give Guisados that rating?	
	0 (Definitely not) - 100 (Enthusiastic)		Correctly Recalled		Reason Included SIG	
	OLS		*logistic*		*logistic*	
	(1)	(2)	(3)	(4)	(5)	(6)
Intercept (Yelp)	52.902***	55.898***	0.665**	0.568	0.061	0.068
	(2.736)	(3.863)	(0.252)	(0.448)	(0.255)	(0.360)
Spoken	−13.003***	−13.014***	1.645**	1.707**	0.685	0.736*
	(3.840)	(3.849)	(0.519)	(0.522)	(0.356)	(0.358)
Both	0.959	0.832	1.107	1.115	−0.156	−0.163
	(3.820)	(3.827)	(0.800)	(0.801)	(0.348)	(0.350)
Grades	7.739***	7.739***	−0.016	−0.006	−0.373***	−0.366***
	(1.034)	(1.036)	(0.095)	(0.096)	(0.106)	(0.106)
Spoken:Grades	3.928**	3.894**	0.150	0.132	−0.166	−0.187
	(1.453)	(1.457)	(0.207)	(0.208)	(0.148)	(0.149)
Both:Grades	0.074	0.122	−0.169	−0.172	0.132	0.135
	(1.453)	(1.456)	(0.308)	(0.308)	(0.143)	(0.144)
Dem. Controls		✓		✓		✓
Observations	957	957	957	957	957	957
R^2	0.252	0.254				
Adjusted R^2	0.248	0.246				
Log Likelihood			−332.002	−328.589	−598.422	−594.624
Akaike Inf. Crit.			676.005	677.177	1,208.844	1,209.248
F Statistic	64.162*** (df = 5; 951)	35.748*** (df = 9; 947)				

Note: *p<0.05; **p<0.01; ***p<0.001
