# Supplementary material for: Conversational technology and reactions to withheld information
Source: PLoS One. 2024 Apr 11;19(4):e0301382. doi: 10.1371/journal.pone.0301382 (PMC11008880; doi:10.1371/journal.pone.0301382)
Supplement: S4 Table — Each column is a different regression model. Standard errors are in parentheses and interactions are indicated by a colon. Regression specifications are: Willingness to dine response regressed on Chat and Spoken indicators.Specification (1) plus controls for age, male, education (1 if > = bachelors), and income (>$75k annually).Outcome variable is an indicator for correctly recalling the SIG (1 if true) with same IV’s as (1).(3) with same IV’s as (2).Outcome variable is an indicator for if a participant mentioned SIG in an open response about their decision regressed on same IV’s as (1).(5) with same IV’s as (2). (PDF) [file pone.0301382.s004.pdf]

	What do you think about going to Guisados?		What was Guisados' [SIG]?		Why did [the participant] give Guisados that rating?	
	0 (Definitely not) - 100 (Definitely)		Correctly Recalled		Reason Included SIG	
	OLS		*logistic*		*logistic*	
	(1)	(2)	(3)	(4)	(5)	(6)
Intercept (Yelp)	79.069***	93.899***	0.299	−0.856	−2.102***	−4.005***
	(2.495)	(5.539)	(0.201)	(0.631)	(0.319)	(0.640)
Chat	−7.137*	−6.490	1.941***	1.979***	1.152**	1.187**
	(3.502)	(3.487)	(0.389)	(0.399)	(0.387)	(0.401)
Spoken	−20.128***	−20.595***	2.484***	2.619***	1.394***	1.597***
	(3.511)	(3.490)	(0.466)	(0.478)	(0.382)	(0.402)
Dem. Controls		✓		✓		✓
Observations	308	308	308	308	308	308
R^2	0.100	0.130				
Adjusted R^2	0.094	0.113				
Log Likelihood			−124.691	−119.463	−161.648	−152.128
Akaike Inf. Crit.			255.381	252.926	329.297	318.256
F Statistic	16.937*** (df = 2; 305)	7.501*** (df = 6; 301)				

Note: *p<0.05; **p<0.01; ***p<0.001
