# Supplementary material for: Conversational technology and reactions to withheld information
Source: PLoS One. 2024 Apr 11;19(4):e0301382. doi: 10.1371/journal.pone.0301382 (PMC11008880; doi:10.1371/journal.pone.0301382)
Supplement: S5 Table — Each column is a different regression model. Standard errors are in parentheses and interactions are indicated by a colon. Regression specifications are: Willingness to dine response regressed on Chat and Spoken indicators interacted with linear grades (A = 4, B = 3).Specification (1) plus demographic controls for age, male, education (1 if bachelors or higher), and income (>$75k annually).Outcome variable is an indicator for correctly recalling the SIG (1 if true) with same IV’s as (1).(3) with same IV’s as (2).Outcome variable is an indicator for if a participant mentioned SIG in an open response about their decision regressed on same IV’s as (1).(5) with same IV’s as (2). (PDF) [file pone.0301382.s005.pdf]

	What do you think about going to Guisados?		What was Guisados' [SIG]?		Why did [the participant] give Guisados that rating?	
	0 (Definitely not) - 100 (Definitely)		Correctly Recalled		Reason Included SIG	
	OLS		*logistic*		*logistic*	
	(1)	(2)	(3)	(4)	(5)	(6)
Intercept (Yelp)	30.458^{**}	33.811^{***}	0.518	0.333	−0.679	−1.181
	(9.514)	(9.778)	(1.051)	(1.118)	(1.264)	(1.322)
Script	20.822	18.040	0.016	0.018	0.314	0.090
	(13.485)	(13.419)	(2.116)	(2.127)	(1.893)	(1.911)
Spoken	26.294	24.435	−1.563	−1.599	−0.781	−0.743
	(13.528)	(13.401)	(1.848)	(1.852)	(1.911)	(1.919)
Grades	13.671^{***}	13.017^{***}	0.044	0.017	−0.224	−0.238
	(2.697)	(2.677)	(0.298)	(0.300)	(0.362)	(0.365)
Chat:Grades	−5.461	−4.620	0.511	0.516	−0.183	−0.131
	(3.818)	(3.797)	(0.614)	(0.617)	(0.545)	(0.549)
Spoken:Grades	−6.456	−5.843	0.814	0.830	0.128	0.114
	(3.839)	(3.803)	(0.541)	(0.543)	(0.546)	(0.549)
Dem. Controls		✓		✓		✓
Observations	597	597	597	597	597	597
R^2	0.072	0.097				
Adjusted R^2	0.064	0.083				
Log Likelihood			−258.601	−257.982	−259.900	−257.951
Akaike Inf. Crit.			529.202	535.965	531.800	535.901
F Statistic	9.120^{***} (df = 5; 591)	7.006^{***} (df = 9; 587)				

Note: $^{*}p<0.05$; $^{**}p<0.01$; $^{***}p<0.001$
