# Supplementary material for: Conversational technology and reactions to withheld information
Source: PLoS One. 2024 Apr 11;19(4):e0301382. doi: 10.1371/journal.pone.0301382 (PMC11008880; doi:10.1371/journal.pone.0301382)
Supplement: S6 Table — Each column is a different regression model. Standard errors are in parentheses and interactions are indicated by a colon. Regression specifications are: Likelihood of hiring service provider response regressed on interaction of Conversational and Withheld indicators.Specification (1) plus controls for age, male, education (1 if > = bachelors), and income (>$75k annually).Outcome variable is an indicator for correctly recalling the availability of a customer reference (1 if true) with same IV’s as (1).(3) with same IV’s as (2).Outcome variable is an indicator for if a participant mentioned customer references in an open response about their decision regressed on same IV’s as (1).(5) with same IV’s as (2). (PDF) [file pone.0301382.s006.pdf]

| | Likelihood of Booking Service Provider | | Was a customer reference available? | | Open response question on reference feature | |
|---|---|---|---|---|---|---|
| | -100 (Extremely Unlikely) to 100 (Extremely Likely) | | Correct Recall of Reference Feature | | Mentioned Reference in Response | |
| | *OLS* | | *logistic* | | *logistic* | |
| | (1) | (2) | (3) | (4) | (5) | (6) |
| Intercept (Visual) | 25.851*** | 45.144*** | −0.121 | −0.737* | −3.738*** | −4.913*** |
| | (3.356) | (7.154) | (0.137) | (0.366) | (0.452) | (0.590) |
| Spoken | 7.971 | 6.396 | 3.187*** | 3.186*** | 2.209*** | 2.290*** |
| | (4.823) | (4.815) | (0.367) | (0.370) | (0.488) | (0.492) |
| Withheld | −10.193* | −10.648* | 1.092*** | 1.088*** | 3.129*** | 3.190*** |
| | (4.880) | (4.842) | (0.211) | (0.214) | (0.477) | (0.480) |
| Spoken:Withheld | −36.869*** | −34.340*** | −2.592*** | −2.606*** | −0.394 | −0.473 |
| | (6.963) | (6.932) | (0.444) | (0.448) | (0.539) | (0.543) |
| Dem. Controls | | ✓ | | ✓ | | ✓ |
| Observations | 801 | 801 | 801 | 801 | 801 | 801 |
| $R^2$ | 0.114 | 0.134 | | | | |
| Adjusted $R^2$ | 0.111 | 0.126 | | | | |
| Log Likelihood | | | −386.782 | −380.226 | −346.741 | −338.790 |
| Akaike Inf. Crit. | | | 781.565 | 776.453 | 701.481 | 693.579 |
| F Statistic | 34.177*** (df = 3; 797) | 17.528*** (df = 7; 793) | | | | |

*Note:* *p<0.05; **p<0.01; ***p<0.001
