# Supplementary material for: Conversational technology and reactions to withheld information
Source: PLoS One. 2024 Apr 11;19(4):e0301382. doi: 10.1371/journal.pone.0301382 (PMC11008880; doi:10.1371/journal.pone.0301382)
Supplement: S2 Data — (ZIP) [file pone.0301382.s009.zip › 2017IRB.pdf]

Carnegie Mellon University

APPROVAL OF SUBMISSION

August 19, 2016

Type of Review:	Modification and Continuing Review
Title of Study:	Human Judgment and Decision-Making
Investigator:<br>Study Team Members:	Alex Imas<br>Shereen Chaudhry<br>Christopher Olivola<br>Jeffrey Galak<br>Talya Lazerus<br>Russell Golman<br>George Loewenstein
IRB ID:	MODCR00000308
Funding:	Name:

The above referenced submission was reviewed by the Carnegie Mellon University Institutional Review Board (IRB) **Full Board** at its **8/9/2016** meeting where it was approved subject to modifications. These modifications were submitted and subsequently **APPROVED on 8/9/2016. This APPROVAL expires on 8/8/2017,** unless suspended or terminated earlier by action of the IRB.

If continuing review approval is not granted before the expiration date of 8/8/2017, approval of this study expires on that date. You can submit a continuing review by navigating to the active study and clicking Create Modification / CR. **Note that submitting for continuing review in a timely manner is the responsibility of the PI.**

The following risk level was determined by the Full Board: **Minimal Risk**.

Unanticipated problems and adverse events must be reported to the IRB within three (3) working days. Any additional modifications to this research protocol or advertising materials pertaining to the study must be submitted for review and granted IRB approval prior to implementation.

The Investigator(s) listed above in conducting this protocol agree(s) to follow the recommendations of the IRB of any conditions to or changes in procedure subsequent to this review. In undertaking the execution of the protocol, the investigator(s) further agree(s) to abide by all CMU research policies including, but not limited to the policies on responsible conduct research and conflict of interest.

Sincerely,

John Zimmerman
IRB Chair
